# Primary Diaphragmatic Dedifferentiated Liposarcoma in a Young Female Patient after Delivery

**DOI:** 10.1155/2016/4042719

**Published:** 2016-05-10

**Authors:** Shinya Sakata, Chieko Yoshida, Sho Saeki, Susumu Hirosako, Hidenori Ichiyasu, Hirotsugu Kohrogi

**Affiliations:** Department of Respiratory Medicine, Kumamoto University Hospital, 1-1-1 Honjo, Chuo-ku, Kumamoto 860-8556, Japan

## Abstract

A 26-year-old woman was admitted with the chief complaint of chest pain. She had delivered her first child 9 months before admission. Computed tomography showed a bulky mass in her left chest, and histopathological analysis revealed it to be dedifferentiated liposarcoma. We initiated doxorubicin chemotherapy, and the tumor mass reduced. After that, we performed vascular embolization along with chemotherapy, but tumor size did not reduce. On the 160th day of illness, the patient died. This is the first report of a primary diaphragmatic dedifferentiated liposarcoma diagnosed after delivery. Establishment of a regimen of chemotherapy for bulky unresectable liposarcoma is necessary.

## 1. Introduction

Intrathoracic liposarcoma is a very rare tumor. About 90%–95% of liposarcomas occur in the retroperitoneum and femoral regions. Liposarcoma does not show predominance in any one sex and occurs most commonly in individuals in their 40s and 50s. Liposarcoma occurring in young patients is rare, and no cases of primary diaphragmatic dedifferentiated liposarcoma diagnosed after delivery have been reported. Here, we present the case of a young 26-year-old woman with primary diaphragmatic dedifferentiated liposarcoma diagnosed after delivery.

## 2. Case Report

A 26-year-old woman was admitted to our hospital with left-sided chest pain and a 4-month history of exertional dyspnea. She had delivered her first child 9 months before admission. Physical examination showed decreased breath sounds in the left lung field. Blood tests showed elevated serum lactate dehydrogenase (343 IU/L) and C-reactive protein (4.28 mg/dL). Serum tumor markers carcinoembryonic antigen (0.5 ng/mL), alpha-fetoprotein (1.9 ng/mL), and soluble interleukin-2 receptor (401 U/mL) were all within normal range.

**Figure 1:**
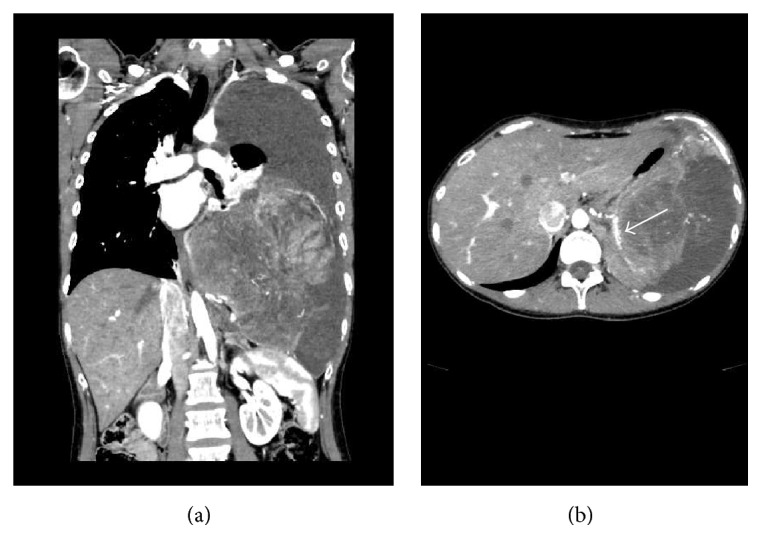
(a) Chest contrast-enhanced computed tomography (CT) showing the bulky intrathoracic mass opacity on the left. (b) The inferior phrenic artery was found to be the main nutrient vessel in the contrast-enhanced CT (arrow).

**Figure 2:**
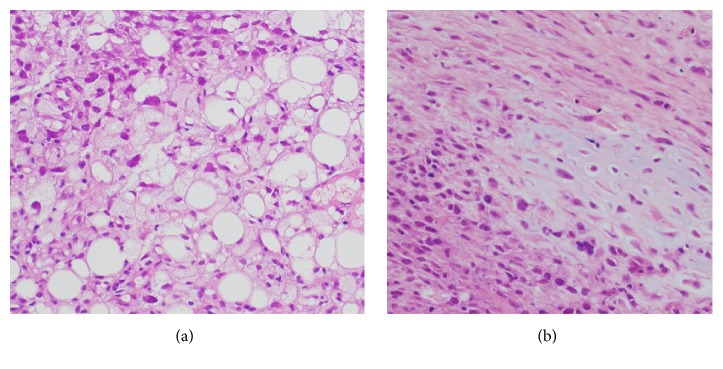
(a) Pathology (hematoxylin and eosin staining, 400x) showing a dense growth of atypical cells indicating differentiation of fat cells; the atypical cells had a clear nucleolus with weakly acidophilic foamy cytoplasm. (b) Pathology (hematoxylin and eosin staining, 400x) showing differentiation of cartilage cells buried among clear cartilage tissues with the dense growth of fusiform immature undifferentiated mesenchymal cells with a high nucleocytoplasmic ratio.

**Figure 3:**
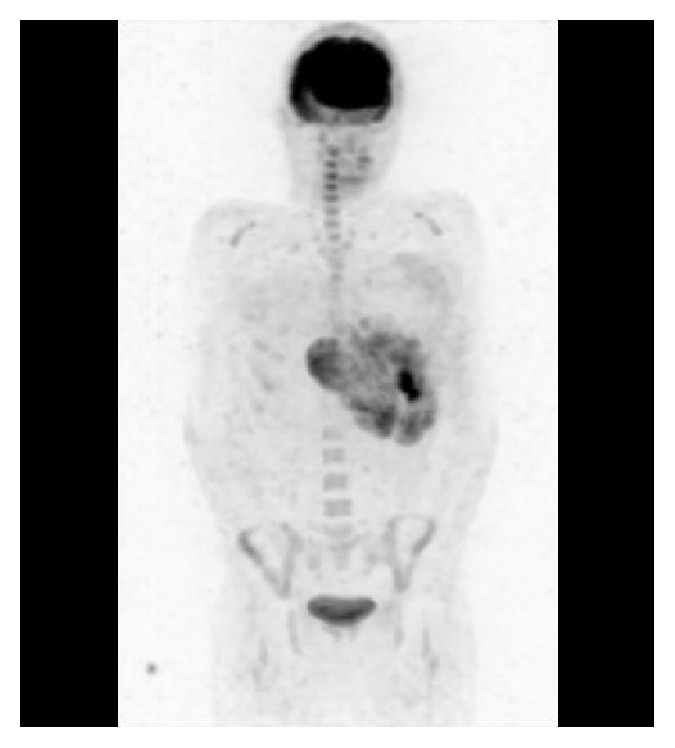
Fluorodeoxyglucose positron emission tomography showing no distant metastases.

Contrast-enhanced computed tomography (CT) showed a bulky mass with heterogeneous enhancement (Figure 1(a)). We performed CT-guided needle biopsy of the mass, and subsequent histopathology revealed dense growth of atypical cells, differentiated fat cells, and atypical cells with a clear nucleolus and weak acidophilic foamy cytoplasm (Figure 2(a)). Histopathology also revealed differentiated cartilage cells buried among clear cartilage tissues with a dense growth of fusiform immature undifferentiated mesenchymal cells with a high nucleocytoplasmic ratio (Figure 2(b)). Thus, we diagnosed dedifferentiated liposarcoma. Because the inferior phrenic artery was the main nutrient vessel of the tumor, we determined the tumor to be of diaphragmatic origin (Figure 1(b)). Fluorodeoxyglucose positron emission tomography showed no distant metastases (Figure 3), and contrast-enhanced magnetic resonance imaging confirmed the absence of brain metastases. Although left pleural dissemination was found by contrast-enhanced chest CT, surgical resection was planned for the purpose of volume reduction. However, circulation was disturbed due to compression of the heart and mediastinum by the bulky tumor mass, leading to postponement of the planned surgical resection.

**Figure 4:**
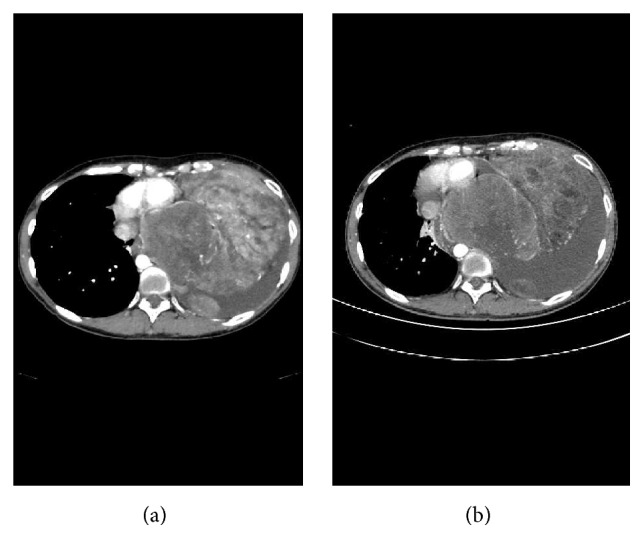
(a) Chest contrast-enhanced computed tomography (CT) before treatment showing a bulky mass in the left chest; pleural dissemination is seen. (b) Chest contrast-enhanced CT after 2 cycles of chemotherapy with doxorubicin showing reduction of the tumor.

Treatment was begun with doxorubicin (25 mg/m^2^, days 1, 2, and 3, every 3 weeks) for tumor reduction, which was obtained after 2 cycles of chemotherapy (Figure 4). Surgical resection was then planned again; however, the tumor showed rapid growth causing congestive heart failure, leading to another postponement of resection. We tried combination chemotherapy with ifosfamide (1200 mg/m^2^, days 1, 2, 3, 4, and 5, every 3 weeks) and doxorubicin (25 mg/m^2^, days 1, 2, and 3, every 3 weeks), but her pulmonary congestion worsened by excessive fluid transfused to prevent ifosfamide toxicity (3750 mL/day of saline), and the treatment was stopped. We next performed vascular embolization of the main nutrient vessel. Subsequently, we tried chemotherapy with single agent of docetaxel (60 mg/m^2^, day 1, every 3 weeks, 1 cycle), doxorubicin (25 mg/m^2^, days 1, 2, and 3, every 3 weeks, 1 cycle), and pazopanib (800 mg/day); however, no treatment effects were observed. On the 160th day of illness, the patient died due to progression of her dedifferentiated liposarcoma.

## 3. Discussion

Intrathoracic liposarcoma is a very rare tumor [1], with 90%–95% occurring in the retroperitoneum and femoral regions [2]. Shmookler and Enzinger reported a frequency of intrathoracic liposarcoma of 2.7% at all sites [3]. Recently, Chen et al. reported 23 cases of intrathoracic liposarcoma. This report included 4 cases (17%) of dedifferentiated liposarcoma, with a median overall survival (OS) of 11 months and a median disease-free survival of 6 months. The report showed inferior OS in dedifferentiated, myxoid, and pleomorphic liposarcomas relative to well-differentiated liposarcomas [4]. In the World Health Organization classification of 2013, liposarcoma can be classified as a histologically intermediate malignant well-differentiated liposarcoma, a histologically malignant myxoid liposarcoma, a pleomorphic liposarcoma, a dedifferentiated liposarcoma, or a liposarcoma not otherwise specified. These classifications are associated with their prognosis [5]. In the present case, a dedifferentiated liposarcoma was mixed with a well-differentiated liposarcoma. Thus, the present case is dedifferentiated liposarcoma of* de novo* development.

Myxoid liposarcoma is the most common type (approximately 50% of all liposarcomas), and the next most common type is well-differentiated liposarcoma. Well-differentiated liposarcoma has a good prognosis, with 5-year survival rates of 80%. Meanwhile dedifferentiated liposarcoma has a poor prognosis, with a recurrence rate of 40%–50%, a metastasis rate of 20%, and a 5-year survival rate of 28% [6, 7]. The dedifferentiated liposarcoma is defined as a high-grade sarcoma that does not form fat and developed from a well-differentiated liposarcoma. In the present case, sites indicating adipogenesis and dedifferentiation were observed by biopsy specimens, respectively. Saito et al. have also reported a case of dedifferentiated liposarcoma of* de novo* development [8].

Small specimens such as those obtained by needle biopsy are insufficient to determine the histologic type of liposarcoma, so tissue diagnosis by surgical resection is preferable. However, in cases where surgical resection is difficult, such as the present case, it is important to biopsy from multiple sites of tumor in order to obtain a correct pathological diagnosis. As a result, we are able to predict a prognosis and a therapeutic effect.

The principle treatment for liposarcoma is surgical resection. Klimstra et al. reported that the mean size of liposarcoma at surgery is 157 mm (range 60–0400 mm) and the mean tumor weight is 1500 g [9]. Surgical resection is typically performed with positive results even on bulky tumors. In one case, long-term survival was reported for a patient with liposarcoma who underwent repeated surgical resection for recurrent disease [10]. In another case, a bulky retroperitoneal liposarcoma could be resected surgically after vascular embolization [11]. In our patient, however, we tried chemotherapy, embolization of the feeding artery, and molecularly targeted medicine; but we were unable to resect the tumor.

There is no established standard regimen of chemotherapy for dedifferentiated liposarcoma. In myxoid liposarcoma, it was reported that chemotherapy with doxorubicin plus ifosfamide had a response rate of 43.2% [12]. For our patient, we tried combination chemotherapy with ifosfamide and doxorubicin, but exacerbation of her pulmonary congestion was induced by the transfusion load administration of ifosfamide. Thus, we were unable to use this chemotherapy regimen. We note that intrathoracic liposarcomas may cause compression of the heart and mediastinum by their bulky mass, making an ifosfamide regimen more difficult in this tumor type than in liposarcomas of other sites of origin. Dedifferentiated liposarcoma has high rate of recurrence. Therefore, the combined modality therapy including the postoperative adjuvant chemotherapy is necessary.

Here, we present a case of dedifferentiated liposarcoma in a young female patient after delivery. Recently, overexpression of the estrogen receptor and androgen receptor in well-differentiated and dedifferentiated liposarcoma was reported [13]. In the present case, the immunohistochemical staining showed positive for progesterone receptor and androgen receptor. An association between pregnancy and dedifferentiated liposarcoma has not been reported. However, the present case exhibited dedifferentiated liposarcoma in a young female patient after delivery. We could not deny the association between these hormone receptors and pregnancy. Thus, a further study is necessary in future. During pregnancy, it is common to not conduct examinations using X-rays to avoid the effects of radiation exposure on the fetus. Therefore, delay of the diagnosis of dedifferentiated liposarcoma is possible, as seen in the present case.

## 4. Conclusion

We present the first report of a primary diaphragmatic dedifferentiated liposarcoma diagnosed after delivery. The current standard of treatment for liposarcoma is surgical resection, which, however, is not always possible in bulky intrathoracic liposarcomas. Therefore, we propose that the establishment of a multidisciplinary regimen for bulky unresectable intrathoracic liposarcomas is necessary.
